# Looking back over 40 years in medical education – the continuing challenge of developing professional artistry and personal educational philosophy

**DOI:** 10.15694/mep.2017.000179

**Published:** 2017-10-10

**Authors:** John Sandars

**Affiliations:** 1 Edge Hill University

**Keywords:** Professional artistry, Personal educational philosophy, Teacher development

## Abstract

This article was migrated. The article was marked as recommended.

Experienced medical educators appear to focus more on their research than developing their professional artistry and personal educational philosophy. This situation has potential major implications for the continuing improvement of teaching and student learning.

## My journey as a medical educator

My first taste of being a medical educator was forty years ago as a junior doctor teaching medical students on the wards. I was determined to put my personal educational philosophy (a fancy term for my deeply held belief and value system about teaching and learning) into practice. My fundamental philosophy, which has been core to my identity as a medical educator, has remained the same over the years but a major challenge has been its refinement and adaptation in response to the wide range of medical education experiences that I have encountered. My personal educational philosophy constantly informs what I do and why I do it that way.

Whilst at school, I had experienced two very contrasting styles of education. The physics teacher was very didactic, with “talk and chalk” accompanied by numerous hand-outs so that we did not need to take notes, but my biology teacher was completely different. In retrospect, he was using problem-based learning, but it was not given such a fancy name at that time, with lots of group work and self-directed tests. Needless to say, my examination performance in biology was far superior to my physics! My first two years at medical school were a traditional “science before clinical” curriculum and I found the didactic nature of the science teaching a major shock to my way of learning! However, when I moved into the clinical years I found the self-directed opportunity to learn the vast knowledge and skills base of the clinical subjects was both liberating and motivating. My examination performance in the clinical subjects was superior to my basic sciences.

My personal educational philosophy was reinforced by my experience as a learner and I had little doubt that the essential role of the teacher was to foster an inquiring mind and self-directed learning. I was also aware of the notion that a teacher cannot make a learner learn - you can only facilitate the learning process. This does not mean that the teacher simply says “find it out for yourself”! The teacher has an essential role to motivate the learner and to facilitate the overall learning process for the learner, with support to identify learning needs and planning to meet these needs, as well as actively supporting the learner to reflect on what they have learned, both in content and the process.

Similar to most medical educators, I initially attended several “train the trainer” courses that were mandatory to teach the increasingly diverse groups of learners that were becoming part of my widening medical educator role, which had a range from medical students and junior doctors to senior doctors attending continuing professional development events. The focus of these training courses was mainly on practical techniques, such as writing learning objectives and constructing assessments. The techniques that I acquired became the basis for my development of professional artistry, where I could begin to skilfully craft my teaching to the needs of the learner and different contexts. I could also begin to appreciate how these techniques might help me to put my personal educational philosophy into daily practice but there was little opportunity to refine and adapt my philosophy.

With an increasing emphasis on delivering and managing medical education in my various educator roles, I decided to enrol in a postgraduate certificate in education. I was the only health professional on this course since its focus was on general adult education. This experience was fantastic, with weekly small group meetings facilitated by tutors who also observed our teaching in practice. The group was expected to read widely and reflect on their practice, with the final assessment being an extensive portfolio of reflections. My professional artistry as a medical educator was developing on an upwards journey and I had the opportunity to grasp the nettle of challenging my deeply held personal educational philosophy, reflecting on how to align my teaching to my beliefs and values.

I was determined to move onto a masters course since my educator role was becoming increasingly academic. The masters course moved the focus of my medical education endeavours in the direction of research. My supervisor, the Professor of Education, was inspirational. I began to read the original sources of the educational theories and philosophies that I had only previously read as a summary in textbooks or review articles, becoming increasingly familiar with the ideas of key educational thinkers, from the learning theories of Bandura and Vygotsky to the pragmatist philosophy of Dewey. The stimulating discussions with my supervisor provided an ideal opportunity to continue the journey of developing my personal educational philosophy but the research project provided little development of my professional artistry as a practical medical educator.

The essential transitional object of a doctoral degree was my next step on the academic pathway and I quickly realised that the focus of my supervision was the academic rigour of my research and dissertation. The research was interesting but I felt that I was no longer on the journey of developing either my professional artistry or my personal educational philosophy. My academic medical educator colleagues rarely discussed how to develop their craft as a teacher and it was even rarer to have vibrant discussions about educational philosophy. The focus of most discussions became increasingly narrowed to “the evidence base” and “quality assurance of teaching”. This situation has continued to a major extent for the last twenty years!

## Take Home Messages

As I reflect on the current state of medical education, I am struck by what appears to be the detachment of most medical educator continuing professional development activities from the reality of teaching and the essential life-long development of professional artistry and personal educational philosophy. Most medical educators are active in teaching yet what appears to be valued are publications and conference presentations instead of discussions between colleagues that are directed at what they actually spend their time doing when they teach. This state of affairs is surprising since school teachers frequently direct their attention to the development of professional artistry and personal philosophy through “teaching circles” which become communities of inquiry. These communities foster a high commitment to changing practice and willingness to try new approaches that are associated with an impact on student learning, with enhanced motivation and improvements in performance ( Stoll et al 2006).

Looking back over 40 years in medical education has highlighted the continuing challenge of developing professional artistry and personal educational philosophy. This development is not an optional extra but is core to being a medical educator after becoming a medical educator through the initial training courses. Undoubtedly research in medical education has informed practice but I am constantly reminded of the original definition of evidence based medicine that clearly emphasises the importance of integrating the evidence with the expertise of the doctor and the views of the patient ( Sackett et al 1996). This definition is often overlooked in medicine and I propose that it is time to revisit it in the era of “evidence based medical education” so that practice is informed by an integration of the findings from research with the teacher’s professional artistry and personal educational philosophy - and of course not forgetting the opinions and wishes of the learner. I have a hunch that medical education of the future could be vastly different if we adopted this stance.

## Notes On Contributors

John is a Professor of Medical Education and qualified in medicine in 1975.
