# Supplementary material for: Looking back over 40 years in medical education – the continuing challenge of developing professional artistry and personal educational philosophy
Source: MedEdPublish (2016). 2017 Oct 10;6:179. [Version 1] doi: 10.15694/mep.2017.000179 (PMC10885301; doi:10.15694/mep.2017.000179)
Supplement: Peer review history

# Reviewer report: Reviewer response for version 1

**DOI:** 10.21956/mep.19355.r28300

**Published:** 2017-10-24

**Authors:** PATRICIA CURY

**Affiliations:** 1 FACERES

This review has been migrated. The reviewer awarded 5 stars out of 5 This is a very important point of view from someone with a very long experience journey in medical education. It shows the continuing challenge of developing professional artistry and personal educational philosophy that we as medical educators should never forget (mainly the youngers), instead of focusing only evidence based medical teaching. Everyone young teacher should read it!

Reviewer Expertise:

NA

No decision status is available

# Reviewer report: Reviewer response for version 1

**DOI:** 10.21956/mep.19355.r28298

**Published:** 2017-10-22

**Authors:** Rehan Ahmed Khan

**Affiliations:** 1 Riphah International University

This review has been migrated. The reviewer awarded 5 stars out of 5 This is a very interesting read by Prof. John Sanders in which he has shared his journey of last 40 years in medical education. One very important point that has been highlighted by him is the value of Education in comparison to research and specifically the role of teacher. This article provides us with food for thought that 'Is research undervaluing education?'. So it is very important to strike a balance between research and education as they complement each other.

Reviewer Expertise:

NA

No decision status is available

# Reviewer report: Reviewer response for version 1

**DOI:** 10.21956/mep.19355.r28302

**Published:** 2017-10-20

**Authors:** P Ravi Shankar

**Affiliations:** 1 American International Medical University

This review has been migrated. The reviewer awarded 4 stars out of 5 Thank you for the opportunity to review this interesting manuscript. I enjoyed reading this well-written manuscript about the journey of a medical educator. I do happen to agree with the author about the increasing gap between medical education research and teaching. Many senior educators concentrate on educational scholarship and may be carrying out little teaching. The traditional ‘science before clinical’ curriculum is now becoming less common and one of its drawbacks is students are often not sure about the clinical importance of the subjects they are learning. I also agree with the author based on my personal experience that small group sessions, problem-based sessions and self-directed learning may be more effective than didactic sessions. Many universities base promotions and tenure decisions on the quality and number of publications. The author’s description of evidence-based practice and evidence-based medical education makes for interesting reading.

Reviewer Expertise:

NA

No decision status is available

# Reviewer report: Reviewer response for version 1

**DOI:** 10.21956/mep.19355.r28297

**Published:** 2017-10-17

**Authors:** sathyanarayanan varadarajan

**Affiliations:** 1 SRM Medical College, SRM University

This review has been migrated. The reviewer awarded 4 stars out of 5 In this Interesting Personal view, Professor John Sandars shared his experiences of 40 years in medical education. He found that the didactic teaching was a major shock to his way of learning during his earlier days of education itself and He felt that the self-directed opportunity to learn the vast knowledge and skills base of the clinical subjects was both liberating and motivating. His personal educational philosophy was reinforced by his experience as a learner and he believed that the essential role of the teacher was to foster an inquiring mind and self-directed learning. He also reflected upon the stimulating discussions with his supervisor provided an ideal opportunity to continue the journey of developing his personal educational philosophy but the research project provided little development of his professional artistry as a practical medical educator. Finally he concludes that practice is informed by an integration of the findings from research with the teacher’s professional artistry and personal educational philosophy alongwith the opinions and wishes of the learner can change the future of medical education...

Reviewer Expertise:

NA

No decision status is available

# Reviewer report: Reviewer response for version 1

**DOI:** 10.21956/mep.19355.r28296

**Published:** 2017-10-12

**Authors:** Nandalal Gunaratne

**Affiliations:** 1 Faculty of Medicine, Wayamba University of Sri Lanka

This review has been migrated. The reviewer awarded 4 stars out of 5 Thank you for sharing your experienced views.Some love to just teach and improve vastly on that competence by adaptation and reflection.Some enjoy research or publish for its gain. Few do both equally well. To recognize that all these people are equally important is what is needed.

Reviewer Expertise:

NA

No decision status is available

# Reviewer report: Reviewer response for version 1

**DOI:** 10.21956/mep.19355.r28301

**Published:** 2017-10-11

**Authors:** Neel Sharma

**Affiliations:** 1 Albert Einstein College of Medicine, Montefiore Medical Center

This review has been migrated. The reviewer awarded 5 stars out of 5 Thanks John for sharing your educational journey! I found it fascinating to read your perspective on PBL. I guess it is truly dependent on the facilitation process and the inspiration that faculty can induce. Maybe I had a bad run of faculty - most were non experts in the subject area and I assume this has something to do with resource availability. Fully agree with the over focus on CV points gathering in medical education. I think we are spending too much time constantly gathering accreditation without focusing on the doing and for me that is enhancing the training at the final year/ F1 phase.

Reviewer Expertise:

NA

No decision status is available

# Reviewer report: Reviewer response for version 1

**DOI:** 10.21956/mep.19355.r28299

**Published:** 2017-10-11

**Authors:** Trevor Gibbs

**Affiliations:** 1 AMEE

This review has been migrated. The reviewer awarded 5 stars out of 5 Thanks John and cannot agree more with your conclusions. Just as we often hear of faculty attaining Certificates / Diplomas and even Masters in Medical Education and then fitting back into the wondrous world of academia, then perhaps we can blame ourselves for probably not following these attainments with longer term, discussive activities about what we do for most of everyday.This of course is not helped by the Universities' measurement of academic quality and reliance upon research and publication

Reviewer Expertise:

NA

No decision status is available
